# Construction and Characterization of a Synergistic lncRNA–miRNA Network Reveals a Crucial and Prognostic Role of lncRNAs in Colon Cancer

**DOI:** 10.3389/fgene.2020.572983

**Published:** 2020-09-15

**Authors:** Bin Zhao, Xiusheng Qu, Xin Lv, Qingdong Wang, Deqiang Bian, Fan Yang, Xingwang Zhao, Zhiwu Ji, Jian Ni, Yan Fu, Guorong Xin, Haitao Yu

**Affiliations:** ^1^Department of Proctology, First Affiliated Hospital of Jiamusi University, Jiamusi, China; ^2^Department of Chemoradiotherapy, First Affiliated Hospital of Jiamusi University, Jiamusi, China; ^3^Department of General Surgery, Samii Medical Center, Shenzhen, China; ^4^Department of Anesthesiology, First Affiliated Hospital of Jiamusi University, Jiamusi, China; ^5^Scientific Research Departments, First Affiliated Hospital of Jiamusi University, Jiamusi, China

**Keywords:** non-coding RNA, synergistic interaction, colon cancer, biological mechanism, prognostic biomarker

## Abstract

Non-coding RNAs such as long non-coding RNAs (lncRNAs) and microRNAs (miRNAs) have been found to be indispensable factors in carcinogenesis and cancer development. Numerous studies have explored the regulatory functions of these molecules and identified the synergistic interactions among lncRNAs or miRNAs, while those between lncRNAs and miRNAs remain to be investigated. In this study, we constructed and characterized an lncRNA–miRNA synergistic network following a four-step approach by integrating the regulatory pairs and expression profiles. The synergistic interactions with more shared regulatory mRNAs were found to have higher interactional intensity. Through the analysis of nodes in the network, we found that lncRNAs played roles that are more central and had similar synergistic interactions with their neighbors when compared with miRNAs. In addition, known colon adenocarcinoma (COAD)-related RNAs were found to be enriched in this synergistic network, with higher degrees, betweenness, and closeness. Finally, we proposed a risk score model to predict the clinical outcome for COAD patients based on two prognostic hub lncRNAs, MEG3 and ZEB1-AS1. Moreover, the hierarchical networks of these two lncRNAs could contribute to the understanding of the biological mechanism of tumorigenesis. For each lncRNA–miRNA interaction in the hub-related subnetwork and two hierarchical networks, we performed RNAup method to evaluate their binding energy. Our results identified two important lncRNAs with prognostic roles in colon cancer and dissected their regulatory mechanism involving synergistic interaction with miRNAs.

## Introduction

Colon adenocarcinoma (COAD) has emerged as one of the leading causes of cancer-related deaths worldwide, with an increasing prevalence (Dienstmann et al., 2015). COAD is a complex disease, whose initiation and progression is closely related with mechanisms of regulation of gene expression (Liu et al., 2017). In recent years, with the development of next-generation sequencing technologies, studies suggested that less than 2% of the human genome encoded protein-coding genes, whereas non-coding RNAs represented most of the human transcriptome (Tay et al., 2014; Levy and Myers, 2016). Non-coding transcripts are divided into various classes, among which long non-coding RNAs (lncRNAs) and microRNAs (miRNAs) have attracted increasing attention. Notably, lncRNAs have been implicated in a diverse range of biological processes, including proliferation, migration, or genomic stability (Mercer et al., 2009; Fatica and Bozzoni, 2014). For instance, some lncRNAs have been reported to regulate gene expression through binding to PRC2 and acting as important controllers of cellular functions (Lee, 2012). miRNAs are small non-coding RNAs that also play a key role in gene regulation (Xu et al., 2019). miRNAs regulate gene expression mainly by binding to the 3′-untranslated regions of mRNAs and leading to their degradation or inhibition, and various studies have demonstrated that aberrant gene expression is closely linked to tumorigenesis, metastasis, and specific tumor stages (Liu et al., 2009).

Previous studies have demonstrated that lncRNAs interact with miRNAs to act on biological traits (Zhang et al., 2019): for example, Kallen et al. (2013) found that the lncRNA H19 modulated miRNA let-7 by performing *in vivo* crosslinking combined with affinity purification experiments. In summary, lncRNAs and miRNAs could interact by regulating mRNAs, thus playing critical roles in the pathological processes involved in disease development (Liao et al., 2019). However, the biological roles and functions of lncRNA–miRNA synergistic interactions have not yet been described in COAD and should be investigated to improve the efficiency of early diagnosis and treatment in the tumorigenesis and progression of this disease.

In this study, we constructed and characterized the lncRNA–miRNA synergistic network involved in COAD by integrating the lncRNA/miRNA–mRNA regulation pairs and the expression profiles of these RNA molecules. In total, we identified 305 positive and 294 negative synergistic lncRNA–miRNA interactions with significantly shared mRNAs. We observed that some of the synergistic lncRNAs and miRNAs were significantly enriched with cancer RNAs, and COAD-related lncRNAs were more important than COAD-related miRNAs. Finally, we proposed a risk score model to predict the clinical outcome of COAD patients based on two prognostic hub lncRNAs, MEG3 and ZEB1-AS1, which were identified by univariate Cox regression analysis. The biological mechanism involving these two lncRNAs was further analyzed. For synergistic lncRNA–miRNA interactions in the hub-related subnetwork and two prognostic lncRNAs related interactions, we provided the total free energy of binding evaluated by RNAup method. Altogether, our analysis provides new insight for exploring the molecular mechanisms of lncRNA–miRNA synergistic interactions and uncovering candidate lncRNA biomarkers for the prognosis of COAD.

## Materials and Methods

### The lncRNA/miRNA–mRNA Regulation Pairs

The miRNA–mRNA target data were downloaded and filtered from StarBase (Li et al., 2014). We chose the miRNA–mRNA interactions which were predicted by at least three of seven target-predicting programs. These seven target-predicting programs included PITA, RNA22, miRmap, DIANA-microT, miRanda, PicTar, and TargetScan. Recent advances in high-throughput sequencing of immunoprecipitated RNAs after cross-linking (CLIP-Seq, HITS-CLIP, PAR-CLIP, CLASH, iCLIP) provide powerful ways to identify biologically relevant RNA-target interactions. To obtain the high-quality miRNA–mRNA datasets, we further selected the miRNA–mRNA interactions which were validated by at least three CLIP-seq data from above interactions as the final miRNA–mRNA interactions. Similarly, we obtained the lncRNA–mRNA interactions that have at least two interactions and supported by at least three CLIP-seq data (Supplementary Table S1). Moreover, we downloaded the experimentally validated lncRNA–mRNA interactions from LncReg and LncRNA2Target databases (Supplementary Table S1; Zhou et al., 2015; Cheng et al., 2019). Integrating the lncRNA–mRNA interactions downloaded from these three databases, we obtained the final lncRNA–mRNA interactions. This way, we obtained 1,336 lncRNA–mRNA and 202,712 miRNA–mRNA high-quality non-redundant interactions.

### Expression Profiles and Clinical Data of COAD Samples

The lncRNA, miRNA, and mRNA expression data for COAD patients were downloaded from the Cancer Genome Atlas (TCGA) database (Tomczak et al., 2015). For each expression profile, RNAs with missing values in more than 30% samples were removed and each of the remaining missing value was imputed by the KNN Imputation. Then all expression values were log2 transformed to obtain the final expression profiles. We chose sample-matched miRNA and lncRNA/mRNA expression profiles for further analysis. The clinical data of COAD patients was also obtained from TCGA.

CpG sites with missing values in more than 30% samples were removed and each of the remaining missing value was imputed by the KNN Imputation.

### Collection of COAD-Related lncRNAs and miRNAs

The COAD-related lncRNAs were downloaded from LncRNADisease (Bao et al., 2019) and lnc2Cancer (Gao et al., 2019). Similarly, we collected the COAD-related miRNAs from several databases, including miR2Disease (Jiang et al., 2009), HMDD (Huang et al., 2019), SM2miR (Liu et al., 2013), and OncomiRDB (Wang et al., 2014).

### Construction of the lncRNA–miRNA Synergistic Interaction Network

To identify the synergistic lncRNA–miRNA interactions, we developed a four-step computational method by integrating lncRNA–mRNA interactions, miRNA–mRNA interactions, and expression profiles of lncRNA, miRNA, and mRNA in COAD samples (Supplementary Figure S1).

First, high-quality lncRNA–mRNA and miRNA–mRNA interactions were downloaded from several databases and processed to obtain the non-redundant data as described above. Second, the regulatory networks of lncRNA–mRNA and miRNA–mRNA were constructed by filtering the lncRNA/miRNA–mRNA pairs obtained from StarBase with their expression profiles. The regulatory correlation between lncRNA/miRNA and mRNA was evaluated by Pearson correlation coefficient based on the matched lncRNA/miRNA and mRNA expression profiles. The pairs with significant correlation were saved as the lncRNA–mRNA (*p*-adjusted < 0.05) and miRNA–mRNA (*R* < -0.4, *p*-adjusted < 0.05) interactions in COAD patients. Third, we identified the co-regulated lncRNA–miRNA pairs by evaluating the significance of their shared regulated mRNAs (hypergeometric-test, *p*-adjusted < 0.01). Fourth, we used Pearson correlation to evaluate the synergistic direction and synergistic power of each co-regulated lncRNA–mRNA pair, and the pairs with *p* < 0.05 were considered the final synergistic lncRNA–mRNA pairs. Finally, after assembling the synergistic lncRNA–mRNA pairs, we obtained the lncRNA–miRNA synergistic interaction network. Two types of nodes were involved in the network (lncRNAs and miRNAs), with positive or negative synergistic regulations.

### Survival Analysis According to the Risk Score Model

We evaluated the clinical outcomes for COAD patients by our risk score model based on the expression levels of MEG3 and ZEB1-AS1. We first divided the COAD samples into training (70% of the samples) and test (30% of the samples) sets. The risk score model was constructed by considering the individual power of MEG3 and ZEB1-AS1 evaluated by the univariable Cox regression analysis and their expression levels in training samples as follows:

$$\mathrm{Risk}\mathrm{score}=\sum_{i=1}^{2}coef_{i}\times exp_{i}$$

where exp*_*i*_* is the expression level of MEG3 or ZEB1-AS1 and coef*_*i*_* is the regression coefficient of MEG3 or ZEB1-AS1 estimated by univariate Cox regression analysis. As a result, the risk score for each COAD patient was computed by the formula:

(1) $$\begin{array}{c} \mathrm{Risk}\mathrm{score}=\left(0.4933\times \mathrm{expression}\mathrm{value}\mathrm{of}\mathrm{MEG3}\right)\\ \;+(1.1077\times \mathrm{expression}\mathrm{value}\mathrm{of}\mathrm{ZEB1}-\mathrm{AS1} \end{array}$$

The median risk score value in the training samples was chosen as the cut-off value to classify patients into high-risk and low-risk groups from the training and test sets, respectively. Survival analyses were performed to assess the difference in clinical outcome between the high-risk and low-risk groups, and statistical significance was evaluated by a log-rank test using the R package “survival.”

### Network Visualization

The networks were visualized by Cytoscape 3.3.0 (Shannon et al., 2003), including the synergistic lncRNA–miRNA network, the hub-related subnetwork and the MEG3/ZEB1-AS1-related hierarchical networks.

## Results

### Construction and Characterization of the Synergistic lncRNA–miRNA Network

**FIGURE 1:**
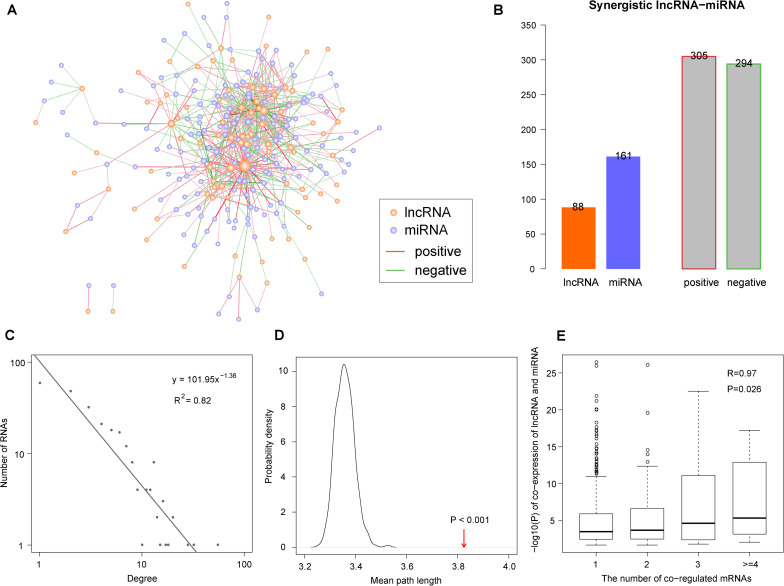
The synergistic long non-coding RNAs (lncRNA)-microRNAs (miRNA) network in the context of colon adenocarcinoma (COAD). **(A)** Global view of the synergistic network. Orange and purple nodes represent lncRNAs and miRNAs, respectively. Red and green edges represent positive and negative synergistic relationships. Larger nodes represent higher degrees. **(B)** Statistics of nodes and edges in the synergistic network. **(C)** Degree distribution of the synergistic network. **(D)** The mean path length of the synergistic network is higher than randomization test. The arrow represents the mean path length in the real network. **(E)** The synergistic lncRNA–miRNA pairs with more co-regulated mRNAs show more significantly synergistic regulatory relationship. The white circles represents the outlier points in boxplots.

Based on the lncRNA/miRNA–mRNA regulation pairs downloaded from databases and expression profiles of lncRNA, miRNA, and mRNA, we constructed a synergistic lncRNA–miRNA network in the context of COAD (Figure 1A). As described in the ‘Materials and Methods’ section, we constructed this network in four steps. In the first step, we obtained 455 lncRNA–mRNA and 28,639 miRNA–mRNA COAD-specific regulation pairs (Table 1). Then, we identified 1,368 co-regulated lncRNA–miRNA pairs with significantly shared mRNAs by a hypergeometric test (Table 1). Finally, Pearson correlation analysis was used to filter the co-regulated lncRNA–miRNA pairs, and we obtained 305 positive and 294 negative synergistic lncRNA–miRNA interactions, covering 88 lncRNA and 161 miRNAs (Table 1, Figure 1B and Supplementary Table S2).

**TABLE 1:** The statistic of COAD specific regulation pairs.

COAD specific pairs	Number of node 1	Number of node 2	Number of edges
lncRNA–mRNA	169 (lncRNA)	313 (mRNA)	455
miRNA–mRNA	289 (miRNA)	6,392 (mRNA)	28,639
Co-regulated lncRNA–miRNA	113 (lncRNA)	194 (miRNA)	1,368
Synergistic lncRNA–miRNA	88 (lncRNA)	161 (miRNA)	305 (positive) 294 (negative)

*long non-coding RNAs (lncRNAs); microRNAs (miRNAs); colon adenocarcinoma (COAD).*

To explore the architecture and features of the synergistic network, its topological properties were analyzed. Through the analysis of node degree distribution, we found that the majority of nodes had few synergistic interactions, while a small portion had many interactions. This fits with the power-law distribution, suggesting that the synergistic lncRNA–miRNA network was scale-free and different from randomly generated networks (*R*^2^ = 0.82, Figure 1C). Moreover, we compared the clustering coefficient between our synergistic network and random networks. The result showed that the lncRNAs and miRNAs in our network had tight synergistic interactions (*p* < 0.001, Figure 1D). The synergistic lncRNA–miRNA pairs share varying numbers of mRNAs. In order to explore the relationship between their synergistic intensity and the number of shared mRNAs, we compared the co-expression significance with different numbers of shared mRNAs. The result showed that those lncRNA–miRNA pairs with more shared regulated mRNAs tended to be more significantly co-expressed to achieve coordinated regulation (*R* = 0.97, *p* = 0.026, Figure 1E).

### lncRNAs Are More Likely to Have Similar Synergistic Interactions With Its Neighbors

**FIGURE 2:**
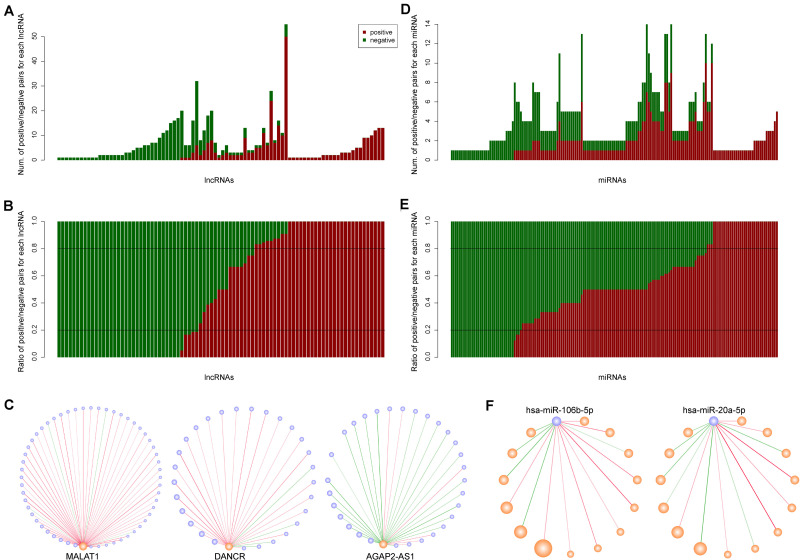
The synergistic interactions between long non-coding RNAs (lncRNAs) and microRNAs (miRNAs). **(A)** The number of positive and negative miRNA neighbors for each lncRNA. Dark red and dark green represent positive and negative interactions, respectively. **(B)** The ratio of positive and negative miRNA neighbors for each lncRNA. **(C)** The synergistic interactions for three lncRNAs: MALAT1, DANCR, and AGAP2-AS1. **(D)** The number of positive and negative lncRNA neighbors for each miRNA. **(E)** The ratio of positive and negative lncRNA neighbors for each miRNA. **(F)** The synergistic interactions for two miRNAs: hsa-miR-106b-5p and hsa-miR-20a-5p.

The lncRNAs and miRNAs in our network had positive and negative synergistic interactions. Therefore, we counted and computed the ratio of positive and negative miRNA neighbors for each lncRNA. The results indicated that each lncRNA had 1∼55 lncRNA neighbors, including 0∼50 positive and 0∼26 negative neighbors (Figure 2A). After calculating the neighbor ratio of each lncRNA, we found that 82.95% of the lncRNAs tended to have the same synergistic direction as most (≥ 80%) of its miRNA neighbors. Among these lncRNAs, 48 and 52% were likely to have positive and negative synergistic interactions with miRNAs, respectively (Figure 2B). Examples of such lncRNAs are MALAT1, DANCR, and AGAP2-AS1 (Figure 2C).

Similarly, we counted and computed the neighbors of each miRNA, and discovered that each miRNA had 1∼14 lncRNA neighbors with 0∼10 positive and 0∼7 negative lncRNA neighbors (Figure 2D). As opposed to lncRNAs, only 43.48% of the miRNAs had the same synergistic direction as most (≥ 80%) of its lncRNA neighbors, while other miRNAs such as hsa-miR-106b-5p and hsa-miR-20a-5p had mixed synergistic relationships with their lncRNA neighbors (Figures 2E,F).

### The Cancer-Related lncRNAs in the Synergistic Network Have Centralized Roles

**FIGURE 3:**
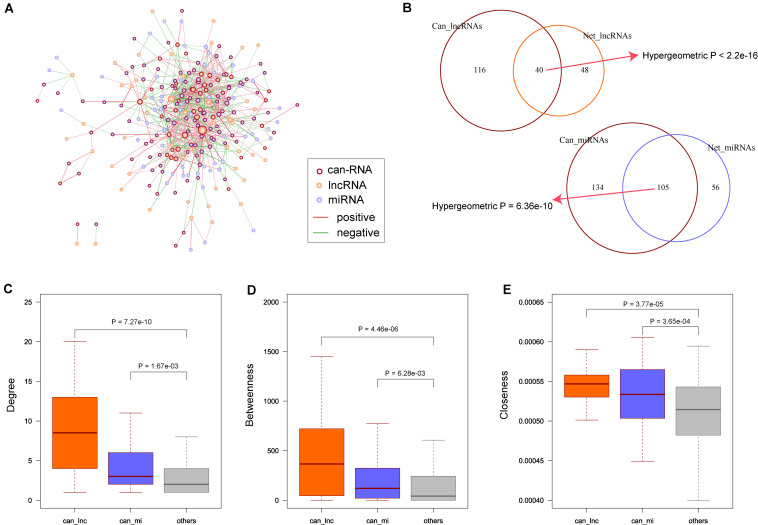
Known colon adenocarcinoma (COAD) related RNAs in synergistic network. **(A)** Known COAD related RNAs were mapping to the synergistic network. Node color and edge color are same as Figure 1A. Nodes with dark red border represent the known COAD related RNAs. **(B)** Enrichment of known COAD related long non-coding RNAs (lncRNAs) and microRNAs (miRNAs) in network. *P-*values are computed by hypergeometric test. **(C)** COAD related lncRNAs and miRNAs have higher degrees than other nodes. **(D)** COAD related lncRNAs and miRNAs have higher betweenness than other nodes. **(E)** COAD related lncRNAs and miRNAs have higher closeness than other nodes. *P*-values are calculated based on Wilcoxon rank sum test.

Many cancer-related genes have been discovered, and to explore the relationship between our synergistic network and COAD, we obtained COAD-related lncRNAs and miRNAs from databases as described in the ‘Materials and Methods’ section. In total, we obtained 116 COAD-related lncRNAs and 134 miRNAs. After mapping these known COAD-related RNAs to our synergistic network (Figure 3A), we found that the synergistic lncRNAs and miRNAs were significantly enriched with the cancer-related RNAs (*p* < 2.2 × 10^–16^ for lncRNAs and *p* = 6.36 × 10^–10^ for miRNAs, Figure 3B). Then, we compared the topological properties of cancer-related RNAs with other RNAs in the synergistic network. The results showed that COAD-related genes had significantly higher degrees, betweenness centrality, and closeness centrality than other nodes in the synergistic network (*p* = 7.27 × 10^–10^, 4.46 × 10^–06^, and 3.77 × 10^–05^ for COAD-related lncRNAs; *p* = 1.67 × 10^–03^, 6.28 × 10^–03^, and 3.65 × 10^–04^ for COAD-related miRNAs; Figures 3C–E). These results suggest that cancer-related lncRNAs and miRNAs have more centralized roles when compared to other RNAs. Moreover, COAD-related lncRNAs appear to be more important than COAD-related miRNAs.

### Identification of Two Central and Prognostic lncRNAs

**FIGURE 4:**
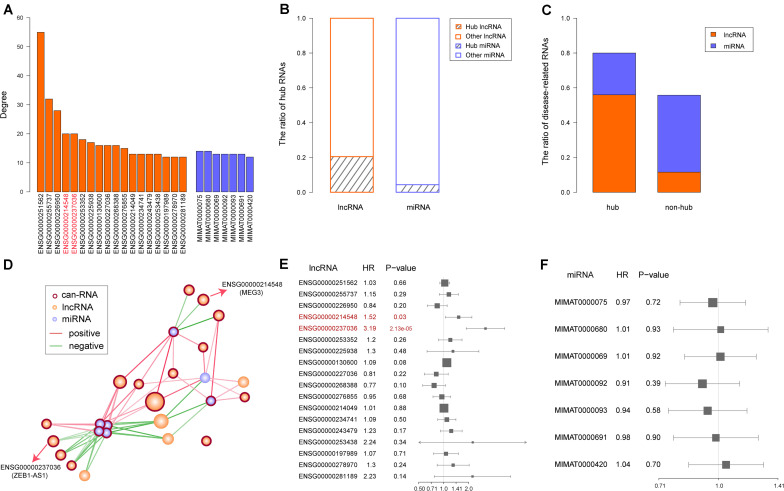
Identification and characterization of hub RNAs. **(A)** Degree of hub RNAs. Orange bars and purple bars represent degree of long non-coding RNAs (lncRNAs) and microRNAs (miRNAs), respectively. **(B)** The ratio of hub and non-hub RNAs in lncRNAs and miRNAs, respectively. **(C)** The ratio of known colon adenocarcinoma (COAD) related lncRNAs and mRNAs in hub and non-hub RNAs. **(D)** Hub subnetwork extracted from the synergistic network. Color of node and edge are same as Figure 3A. **(E)** The result of univariable Cox regression for each hub lncRNA. The prognostic lncRNAs are marked in red. **(F)** The result of univariable Cox regression for each hub miRNA.

Numerous studies have reported hub genes as playing key roles in cancer (Chen et al., 2017; Das et al., 2017; Yin et al., 2019). To identify the hub lncRNAs and miRNAs in our synergistic network, we computed a degree for each node and sorted them in a descending order. Then, we chose 10% of the nodes with the highest degrees as the hub RNAs, including 18 lncRNAs and seven miRNAs (Figure 4A). When comparing the ratio of hub lncRNAs among all lncRNAs with the ratio of hub miRNAs among all miRNAs, we found that a greater ratio of lncRNAs were identified as the hub RNAs (20% *vs.* 4%, Figure 4B), suggesting important roles of lncRNAs in the synergistic network and in accordance with our previous results. Moreover, we found that the majority of the hub RNAs were known COAD-related RNAs, and this ratio was higher than that in non-hub RNAs (80% *vs.* 56%, Figure 4C). The COAD-related hub RNAs included 18 lncRNAs and seven miRNAs. Next, we extracted the edges that connected two hub RNAs and found that all hub RNAs were connected, except for one lncRNA: UCA1. The hub subnetwork is depicted in Figure 4D. In accordance with the synergistic network described above, we found that the lncRNAs and miRNAs in the hub subnetwork were likely to interact with their neighbors in similar and different directions, respectively (Figure 4D).

**FIGURE 5:**
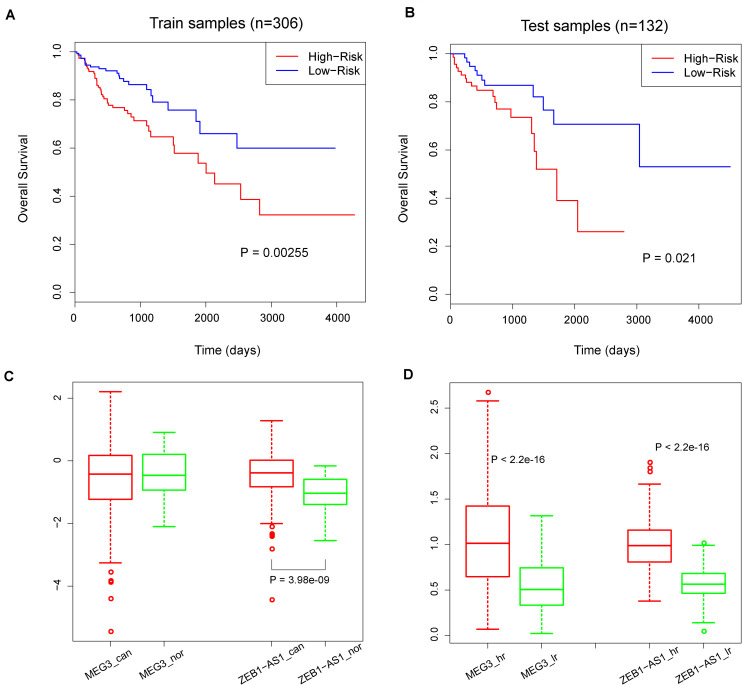
**(A)** Kaplan–Meier curves of overall survival in high-risk and low-risk groups in training set. **(B)** Kaplan–Meier curves of overall survival in high-risk and low-risk groups in test set. *P*-values were calculated by the log-rank test. **(C)** The colored boxes and circles represents expression values of MEG3 and ZEB1-AS1 in cancer and normal samples. ‘can’ represent ‘cancer’ and ‘nor’ represent ‘normal’. **(D)** The colored boxes and circles represents expression values of MEG3 and ZEB1-AS1 in high-risk and low-risk samples. ‘hr’ represent ‘high-risk’ and ‘lr’ represent ‘low-risk’. *P*-values are calculated by Wilcoxon rank sum test.

Considering that hub RNAs have key roles in the development of cancer, in our next step we evaluated the prognostic ability of each hub RNA. Through univariate Cox regression analysis, we identified two risk lncRNAs in COAD patients, MEG3 and ZEB1-AS1 (HR = 1.52 and *p* = 0.03 for MEG3, HR = 3.19 and *p* = 2.13 × 10^–05^ for ZEB1-AS1, Figures 4E,F). Furthermore, we combined these two lncRNAs using the risk score model to predict the clinical outcome of COAD patients. The risk score model was constructed according to Equation 1, with 0.4933 as the regression coefficient for MEG3 estimated by the univariate Cox regression analysis, and 1.1077 as the regression coefficient for ZEB1-AS1. The median risk score of training samples was used as the cut-off value (1.22) to separate the high-risk and low-risk groups. Survival analysis revealed a significant difference in overall survival between these two groups (log-rank test *p* = 0.00255, Figure 5A). Furthermore, we computed the risk score of samples in the test set based on the risk score model and divided these samples into high-risk and low-risk groups. Comparing the clinical outcome of samples between the two groups, we found that low-risk samples in the test set also showed significantly better prognosis (log-rank test *p* = 0.021, Figure 5B). In addition, we randomly selected different sample sets (70∼90% of all COAD samples and the 70∼90% of the test samples), computed their risk scores, and compared the survival difference between high-risk and low-risk groups. The results showed that the risk score model could predict the clinical outcome of COAD patients (Supplementary Figure S2). This result illustrated the robust prognostic ability of the MEG3 and ZEB1-AS1 lncRNAs.

To explore the roles of MEG3 and ZEB1-AS1 during cancer progress, we compared the expression values of MEG3 and ZEB1-AS1 in cancer samples and normal samples. As a result, we found that MEG3 presented similar expression levels in both contexts (*p* = 0.66), while ZEB1-AS1 showed a significantly higher expression in cancer samples (*p* = 3.983 × 10^–09^, Figure 5C). In addition, we compared the expression levels of these two lncRNAs in high-risk and low-risk samples: results showed that MEG3 and ZEB1-AS1 had significantly higher expression values in high-risk samples (all *p* < 2.2 × 10^–16^, Figure 5D). We also downloaded two GEO lncRNA expression datasets associated with colon diseases, including GSE77013 and GSE67106 (Mirza et al., 2015; Padua et al., 2016). The expression of MEG3 and ZEB1-AS1 were found to be highly expressed in disease samples compared with control samples (*t*-test, *P* = 0.09, 0.016, and 1.23e-08, Supplementary Figure S3). Due to the lack of ZEB1-AS1 probes in GSE77013, we didn’t compare its expression values between disease and control samples. These results demonstrate that the high expression of these two lncRNAs is associated with colon disease. Summarizing these results, we believe that MEG3 played a role in cancer development while ZEB1-AS1 could act in both carcinogenesis and cancer development. These results were consistent with previous studies (Fu and Cui, 2017; Gong et al., 2017; Liu et al., 2019; Hu et al., 2020; Ni et al., 2020).

### Hierarchical Networks for Elucidating the Biological Mechanism of MEG3 and ZEB1-AS1

**FIGURE 6:**
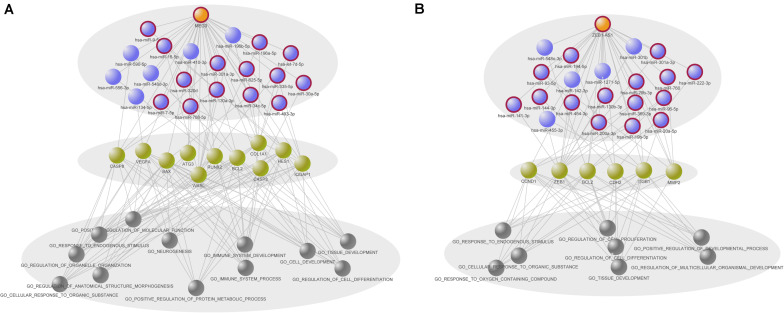
Two hierarchical models of prognostic long non-coding RNAs (lncRNAs). **(A)** Hierarchical model of MEG3 related synergistic regulatory interactions. **(B)** Hierarchical model of ZEB1-AS1 related synergistic regulatory interactions. The models are exhibited hierarchically by three levels, including synergistic lncRNA–microRNAs (miRNA) interactions, regulatory mRNAs and annotated gene ontology (GO) biological processes of mRNAs. GO terms annotated to at least seven mRNAs and five mRNAs are showed in **(A,B)**, respectively. Orange, purple and green nodes represent lncRNAs, miRNAs and mRNAs, respectively. Gray nodes represent the GO terms.

To contribute to the understanding of the synergistic interactions of MEG3 and ZEB1-AS1, we proposed hierarchical models to systematically illustrate the regulatory process. As shown in Figure 6, MEG3 or ZEB1-AS1 regulate mRNAs by synergistic interactions with miRNAs, and further participate in cancer biological processes. Among the miRNAs that have synergistic interactions with MEG3, 14 out of 20 miRNAs were known COAD-related miRNAs. These miRNAs, along with MEG3, further regulate 11 mRNAs, including CASP3, CASP8, and vascular endothelial growth factor (VEGFA). Previous studies have indicated that polymorphisms in CASP3 and CASP8 are related to colon cancer (Goodman et al., 2006; Choi et al., 2012), and the VEGFA was also significantly associated with rectal cancer (Slattery et al., 2014). In this study, we uncovered their upstream regulatory factors, which were MEG3 and its synergistic miRNAs. This result might be another indication of the roles of these mRNAs in carcinogenesis. Through the integration of annotation information on mRNAs, we found that MEG3 and its synergistic miRNAs were mainly associated with cancer-related processes such as immune system development, cell development, tissue development, cell differentiation, protein metabolism, and other processes.

Regarding the synergistic miRNAs related to ZEB1-AS1, 15 out of 20 were found to be known COAD-related miRNAs. ZEB1-AS1 and these miRNAs synergistically regulated six genes, such as CyclinD1 (CCND1) and zinc finger E-box binding homeobox 1 (ZEB1). CCND1 is a key cell cycle regulatory protein and its polymorphism has been found to be significantly associated with overall COAD risk (Xie et al., 2017). Li et al. (2012) reported that IL-1β may promote colon tumor growth and invasion through the activation of colon cancer stem cell self-renewal and epithelial-mesenchymal transition (EMT), and ZEB1 plays a critical role in these two processes. Moreover, we observed that the mRNAs regulated by ZEB1-AS1 and its synergistic miRNAs were annotated to cancer-related gene ontology (GO) terms such as cell differentiation, cell proliferation, and developmental processes.

In light of these results, we believe that MEG3 and ZEB1-AS1 play important roles in the initiation and progression of colon cancer, through their synergistic interactions with cancer-related miRNAs and finally regulating cancer-related mRNAs that were associated with cancer biological processes. Our results could contribute to the understanding of important roles of synergistic lncRNA–miRNA interactions in tumorigenesis, expand the complexity of the ncRNA–mRNA regulatory network, and provide potential therapeutic targets for colon cancer treatment.

## Discussion

Colon adenocarcinoma is the third most common cancer worldwide and has become a widespread health issue for its highly mortality and morbidity (Bertotti et al., 2015; Marmol et al., 2017). Recent studies suggested that interactions between lncRNAs and miRNAs in the regulation of mRNA expression played important regulatory roles in the initiation and progression of COAD (Yu et al., 2017). However, the regulatory mechanisms through which lncRNA–miRNA interactions are involved in the progression of this disease are stil unclear.

Long non-coding RNAs–miRNA synergistic interactions are critical for many biological functions and exploring these interactions contributes to a further understanding of the process of tumorigenesis and development of COAD (Guil and Esteller, 2015; Wang et al., 2019a). More importantly, increasing evidence shows that the lncRNA–miRNA interaction network is implicated in several pathophysiological processes, including human cancers (Lin et al., 2019). In this work, we constructed and characterized the lncRNA–miRNA synergistic network by integrating lncRNA–mRNA interactions, miRNA–mRNA interactions, and the expression profiles of lncRNA, miRNA, and mRNA in COAD samples. The analysis of this synergistic network allowed the detection of complicated features and functions of RNA regulatory interactions and how lncRNAs and miRNAs could play regulatory roles in the tumorigenesis and progression of COAD (Wang et al., 2020). Our results indicated that the synergistic lncRNAs and miRNAs were significantly enriched with cancer-related RNAs. In addition, COAD-related lncRNAs and miRNAs had significantly higher degrees, betweenness centrality, and closeness centrality than other nodes in the synergistic network. Further analysis showed that cancer-related miRNAs, especially lncRNAs, had more centralized roles when compared with other RNAs. Altogether, our study of lncRNA–miRNA interactions could contribute with crucial information in the understanding of the regulatory mechanisms through which ncRNAs act, as well as with the identification of molecular targets for the diagnosis and treatment of COAD.

Reliable prediction of RNA–RNA binding energies is crucial. RNAup is an effective method, which involved two energy contributions, including (1) the energy necessary to ‘open’ the binding site and (2) the energy gained from hybridization. To improve the medical effectiveness of our results, we performed RNAup to compute the potential binding possibility between lncRNAs and miRNAs. The sequence of lncRNA transcripts and miRNA were downloaded from Ensemble and miRBase database, respectively (Supplementary Table S3; Kozomara et al., 2019; Yates et al., 2020). For interactions in the hub-related subnetwork and two hierarchical networks, we provided the total free energy of binding for each lncRNA–miRNA interaction (Supplementary Tables S4, S5). Based on the total free energy of binding we provided, users can acquire both direct and indirect interactions by their own cutoffs. This way, we expect to provide results that have higher potential medical usefulness.

Accumulating evidence revealed that lncRNAs acted as prognostic biomarkers and regulated cell functions in colorectal cancer (Yin et al., 2015; Wang et al., 2019b). Through analyses of node degree and univariate Cox regression analysis, we identified two important lncRNAs: MEG3 and ZEB1-AS1. We further proposed two hierarchical models to systematically illustrate the regulatory process of these two lncRNAs. In the hierarchical models, most miRNAs which have synergistic interactions with MEG3 or ZEB1-AS1 were found to be known COAD-related miRNAs. Moreover, we found that some mRNAs regulated by the lncRNAs and miRNAs were reported to be associated with COAD. Our results proposed another indication of the roles of these mRNAs in carcinogenesis. We believe that other ncRNAs and mRNAs in the hierarchical models were also COAD-related RNAs. Our results provide potential therapeutic targets for colon cancer treatment. Finally, we proposed a risk score model to predict the clinical outcome of COAD patients and demonstrated the utility of lncRNAs as promising biomarkers.

## Data Availability Statement

All datasets generated in this study are included in the article/Supplementary Material.

## Author Contributions

HY conceived and designed the experiments. BZ, XQ, XL, QW, DB, FY, XZ, ZJ, JN, GX, and YF collected and analyzed data. HY and BZ wrote this manuscript. All authors read and approved the final manuscript.

## Conflict of Interest

The authors declare that the research was conducted in the absence of any commercial or financial relationships that could be construed as a potential conflict of interest.
