# Supplementary material for: Construction and Characterization of a Synergistic lncRNA–miRNA Network Reveals a Crucial and Prognostic Role of lncRNAs in Colon Cancer
Source: Front Genet. 2020 Sep 15;11:572983. doi: 10.3389/fgene.2020.572983 (PMC7522580; doi:10.3389/fgene.2020.572983)
Supplement: Supplementary file 1 [file Data_Sheet_1.PDF]

Supplemental files for "Construction and characterization of a synergistic lncRNA-miRNA network reveals a crucial and prognostic role of lncRNAs in colon cancer"

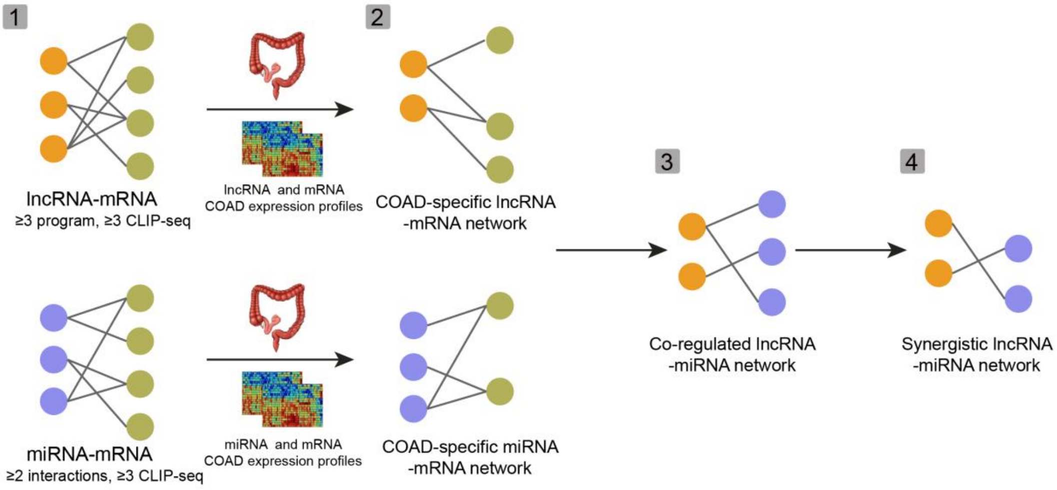

Figure S1. The workflow of constructing synergistic lncRNA-miRNA network. We performed a four-step method to construct the synergistic lncRNA-miRNA network. Step 1, we downloaded the lncRNA-mRNA interactions and miRNA-mRNA interactions from several databases. Step 2, we obtained the COAD-specific lncRNA-mRNA and miRNA-mRNA networks based on the interactions obtained from step 1 and the expression profiles of lncRNA, miRNA and mRNA. Step 3, the co-regulated lncRNA-miRNA network was constructed by hypergeometric-test. Step 4, the co-regulated and co-expressed lncRNA-miRNA interactions were identified and we constructed the synergistic lncRNA-miRNA network.

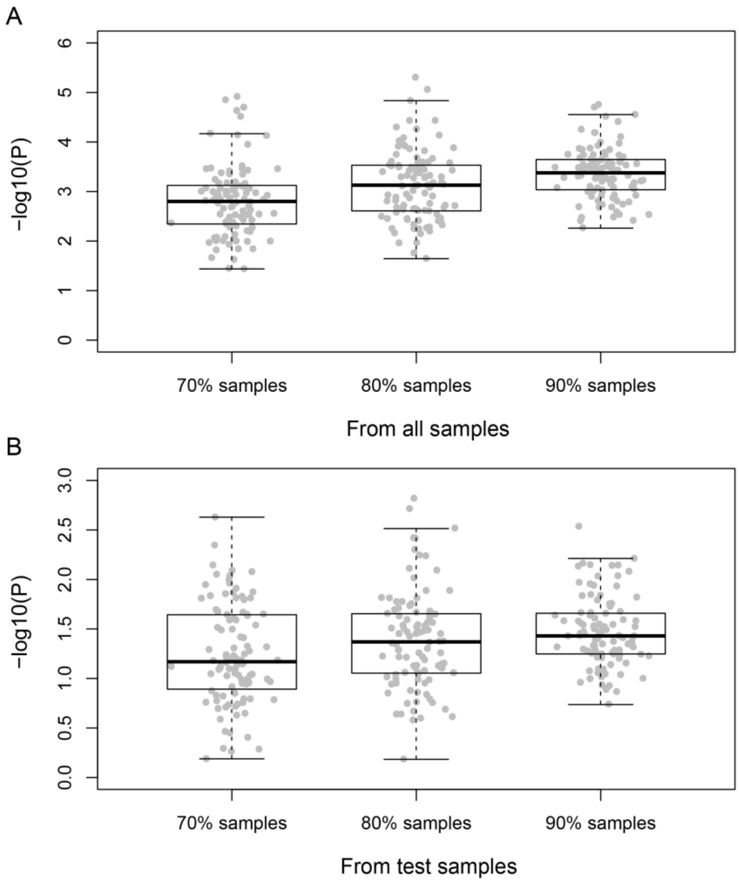

Figure S2. –log10(P) of the random samples selected from (A) all samples and (B) only test samples. P values represent the log-rank result of two group samples divided by risk-score model.

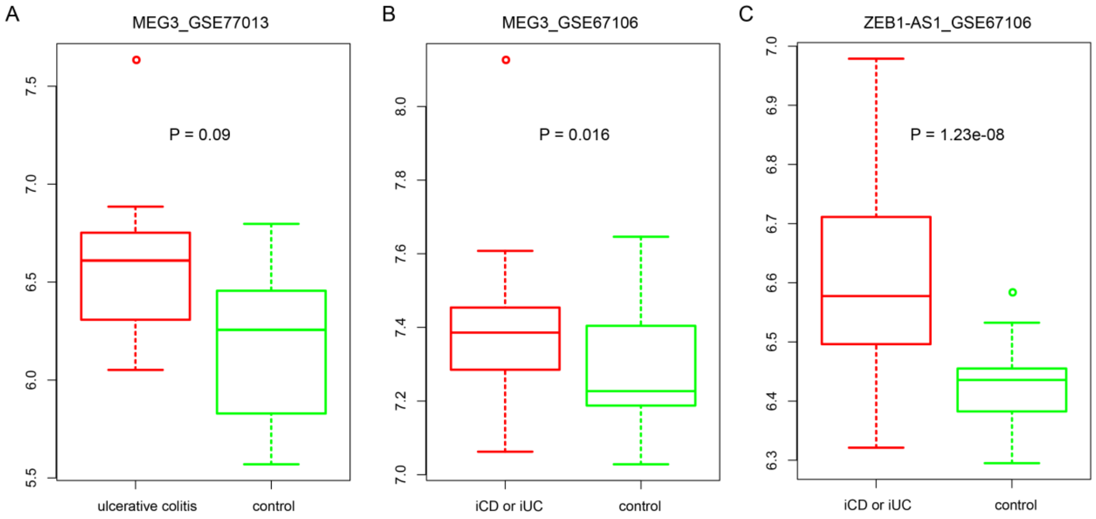

Figure S3. The expression of MEG3 and ZEB1-AS1 in validation datasets. The datasets are (A) GSE77013 (B and C) and GSE67106. P values are computed by *t-test*.

Table S1. The lncRNA-mRNA and miRNA-mRNA interactions downloaded from StarBase. (Table S1.xlsx)

Table S2. The synergistic lncRNA-miRNA network. (Table S2.xlsx)

Table S3. The sequence of lncRNAs and miRNAs in the hub-related subnetwork and two hierarchical networks. (Table S3.xlsx)

Table S4. The potential binding possibility between lncRNAs and miRNAs in the hub-related network. (Table S4.xlsx)

Table S5. The potential binding possibility between lncRNAs and miRNAs in two hierarchical networks. (Table S5.xlsx)
