# Supplementary material for: Construction and Characterization of a Synergistic lncRNA–miRNA Network Reveals a Crucial and Prognostic Role of lncRNAs in Colon Cancer
Source: Front Genet. 2020 Sep 15;11:572983. doi: 10.3389/fgene.2020.572983 (PMC7522580; doi:10.3389/fgene.2020.572983)
Supplement: Supplementary file 2 [file Table_1.DOCX]

**Table 1. The statistic of COAD specific regulation pairs.**

| COAD specific pairs | Num. of node 1 | Num. of node 2 | Num. of edges |
| --- | --- | --- | --- |
| lncRNA-mRNA | 169 (lncRNA) | 313 (mRNA) | 455 |
| miRNA-mRNA | 289 (miRNA) | 6392 (mRNA) | 28639 |
| Co-regulated lncRNA-miRNA | 113 (lncRNA) | 194 (miRNA) | 1368 |
| Synergistic lncRNA-miRNA | 88 (lncRNA) | 161 (miRNA) | 305 (positive)  294 (negative) |
